# Forecasting Faces in the Cortex: Comment on ‘High-Level Prediction Signals in a Low-Level Area of the Macaque Face-Processing Hierarchy’, by Schwiedrzik and Freiwald, *Neuron* (2017)

**DOI:** 10.1016/j.tics.2017.12.001

**Published:** 2018-02

**Authors:** Lucy S. Petro, Lars Muckli

**Affiliations:** 1 Centre for Cognitive Neuroimaging, Institute of Neuroscience and Psychology, College of Medical, Veterinary and Life Sciences, University of Glasgow, 58 Hillhead Street, Glasgow, G12 8QB, UK

## Abstract

Although theories of predictive coding in the brain abound, we lack key pieces of neuronal data to support these theories. Recently, Schwiedrzik and Freiwald found neurophysiological evidence for predictive codes throughout the face-processing hierarchy in macaque cortex. We highlight how these data enhance our knowledge of cortical information processing, and the impact of this more broadly.

Despite having no direct access to the world, neuronal networks must ultimately represent useful information about the external environment. Fortunately, our prior experiences are stored as information in the brain. If the brain can use this stored information to generate a prediction that matches the current environment, then the neuronal microcircuit has represented something useful about the world. This hypothetical model of brain function is conceptualized by predictive coding [1].

Predictive coding theories state that neurons generate predictions higher up in the cortical hierarchy, and test these predictions against incoming sensory information in lower areas, producing an error signal if there is a mismatch between the prediction and current sensory information [1]. This theory, whereby an internal testing mechanism flows down the cortical hierarchy, could support a range of brain functions including social cognition. Consider recognizing the face of a friend from a distance: your brain might predict that the face of the accompanying person is that of his or her partner. Such context-dependent expectations are essential for successful social behavior.

However, there was no prior neuronal evidence that prediction error signals generated in a lower area tested the prediction of a higher area – a hallmark stipulated by predictive coding. In a recent paper, Schwiedrzik and Freiwald [2] revealed how prediction-generating and prediction-testing neurons in the macaque face network exchange codes until predictions match the input. The neuronal guessing-game starts with a template generated from memory that neuronal processes then test against sensory evidence lower in the cortical hierarchy. The macaque face network is an attractive system for scrutinizing prediction principles. Face-selective cortical patches form an interconnected hierarchical network but have different and specific functional preferences for faces. Therefore, we have a hypothesis about the prediction that each patch generates and to which patch it will communicate this prediction.

During a learning phase, the authors presented sequential face image pairs to monkeys, training them to expect a particular ‘successor’ image after seeing the first ‘predictor’ image. Images differed on dimensions to which face patches are tuned (head orientation or identity). The authors later manipulated the order of images, inducing an error response in neurons in the ‘ML’ (middle lateral) patch because the new face pairs violated the monkey’s expectation along dimensions of identity, viewpoint, or both. By comparing ML responses to the successor image under predictable and unpredictable conditions, the authors showed that prediction errors in ML reflect identity specificity and view invariance.

Importantly, the prediction error properties are not those that ML is tuned to – but are instead the properties of higher areas. This suggests that ML constructs the error from the comparison with top-down predictions, a mechanism by which a lower area inherits the features of higher areas. This contrasts with previous accounts of top-down feedback that conceptualize the recovery of high-resolution features by probing lower-hierarchy visual areas [3]. The authors also confirm the behavioral relevance of this neuronal process. A separate, near-identical experiment revealed that humans detected faces faster in predictable versus unpredictable conditions.

This study provides evidence of predictive signaling in a brain system where information- processing determinants are well established. The data encourage the re-evaluation of cortical processing strategies. The currency which processing might trade in is the violation of a prediction, a signal that the cortex broadcasts upstream to higher areas until those areas generate a better hypothesis that matches the input. Thus, cortical communication might be well conceptualized as a recurrent negotiation loop attempting to resolve prediction error. This model fits well for perception in which higher-order representations are well constrained and predict precise sensory inputs; here we can estimate what an error-detecting neuron might be tuned to. However, we do not know whether or how prediction sustains processing for a sensory input that is not immediately available but will arrive in the future. This long-term mental forecasting, for example planning your route home, involves complex and abstract internal representations that unfold over longer durations than sensory signaling.

Another challenge is to understand the precise neuronal machinery for predictive processing. The current data suggest that cortical feedback carries predictions. Distal dendrites in upper layers of mammalian cortex are a target of feedback and could offer a means by which individual neurons test and compare feedforward and feedback signals [4], potentially resolving the error. It is a challenge, however, to decode the language of feedback, in contrast to feedforward responses that are driven directly by the stimulus. In as much as feedforward processing generalizes across viewpoints (e.g., when identifying a face), feedback projections might inherit viewpoint invariance through a quick succession of predictions of mirror-symmetric alternatives of the same identity. We also find this generalization of predicted features in feedback projections to primary sensory areas. A scene remains predicted by the top-down projection even if the spatial frequency band of the input changes [5]. Complex top-down predictions may not only allow contextualization but also a rapid reduction of prediction errors. A neuron therefore signals not only the presence of a feature but also whether this feature was unexpected, and when this surprise is resolved. Interestingly, the current data also do not necessarily imply a complex translation of one type of representational format into another, but that predictions can be communicated on the backbone of straightforward wiring properties.

Schwiedrzik and Freiwald’s data are a striking demonstration that visual neurons perform predictive processing. Evidence of neural prediction marks a paradigm shift that engages theorists and empirical scientists in psychology, neuroscience, and philosophy. Philosophical themes being redefined using neurocomputational rules of prediction include conscious experience and embodied cognition (e.g. [6]). Algorithmic developments in artificial intelligence, brain-inspired computing, and robotics are rooted in neuronal prediction (e.g., [7]). Deficient predictive processing might contribute to psychotic symptoms [8] and neurodevelopmental disorders such as autism [9]. Proponents of predictive processing argue we can explain such data most parsimoniously in the framework of prediction. Others argue we have insufficient empirical evidence to substantiate predictive processing. Ultimately, theoretical frameworks will need to adjust to empirical data before we can model precisely how neurons predict or how prediction supports the full range of brain functions. Similar to the pioneering finding of Hubel and Wiesel of feature detectors in visual cortex [10], Schwiedrzik and Freiwald found prediction error-detection signals in the face-processing network. Such data are essential if prediction is to transform from a conceptual framework into a measurable and general mechanism of brain function.
